# Supplementary material for: Effects of Non-Essential Amino Acids on Knee Joint Conditions in Adults: A Randomised, Double-Blind, Placebo-Controlled Trial
Source: Nutrients. 2022 Sep 2;14(17):3628. doi: 10.3390/nu14173628 (PMC9460309; doi:10.3390/nu14173628)
Supplement: Supplementary file 1 [file nutrients-14-03628-s001.zip › nutrients-1831259-supplementary.pdf]

Supplementary Material

Table S1. The results of safety evaluations at W0, W4 and W12.

	W0		W4		W12	
	**6AA**	**Placebo**	**6AA**	**Placebo**	**6AA**	**Placebo**
BMI (kg/m²)	22.0 (2.5)	22.6 (2.8)	22.1 (2.5)	22.6 (2.7)	22.2 (2.6)	22.7 (2.9)
SBP (mmHg)	120.6 (11.6)	121.1 (11.5)	121.4 (11.6)	122.1 (9.7)	122.4 (9.8)	120.2 (10.2)
DBP (mmHg)	73.3 (7.8)	74.4 (10.2)	73.4 (8.3)	75.9 (9.5)	74.8 (7.6)	74.8 (8.9)
Pulse rate (bpm)	69.2 (10.0)	68.1 (9.5)	67.7 (7.8)	69.2 (7.5)	69.6 (8.0)	70.0 (7.7)
WBC (μL)	5148 (1514.4)	4956 (1151.5)	5164 (1394.9)	4660 (1209.3)	5412 (1851.2)	4832 (1171.1)
RBC (×10⁴/μL)	443.5 (33.9)	446.6 (57.5)	449.7 (35)	459.6 (53.5)	458.6 (37.5)	455.9 (49.6)
Hb (g/dL)	13.3 (1.0)	13.5 (1.7)	13.5 (1.0)	14.0 (1.6)	13.7 (1.1)	13.8 (1.6)
Ht (%)	42.0 (3.0)	42.5 (5.0)	43.3 (3.2)	44.1 (4.5)	43.9 (3.3)	43.8 (4.2)
Platelet count (10⁴/μL)	28.3 (6.1)	26.8 (6.2)	29.5 (6.6)	27.3 (5.7)	29.4 (6.5)	28.2 (6.4)
Total protein (g/dL)	7.0 (0.3)	7.1 (0.4)	7.1 (0.4)	7.3 (0.4)	7.2 (0.4)	7.4 (0.4)
Albumin (g/dL)	4.3 (0.2)	4.3 (0.2)	4.5 (0.2)	4.5 (0.3)	4.4 (0.3)	4.5 (0.3)
Total bilirubin (mg/dL)	0.7 (0.3)	0.8 (0.3)	0.7 (0.2)	0.8 (0.2)	0.7 (0.2)	0.8 (0.3)
AST (U/L)	18.6 (4.6)	20.3 (5.1)	19.2 (7.4)	21.1 (6.0)	18.9 (5.6)	22.5 (8.7)
ALT (U/L)	16.0 (8.9)	16.2 (6.6)	17.6 (11.4)	16.8 (8.3)	18.1 (9.5)	17.9 (9.2)
LDH (U/L)	176.1 (33.8)	188.3 (26.3)	176.2 (23.8)	190.2 (22.5)	172.4 (31.9)	187.4 (29.3)
ALP (U/L)	62.1 (20.7)	63.3 (16.2)	62.5 (24.2)	64.8 (14.9)	64.0 (23.8)	64.7 (16.6)
γGTP (U/L)	19.6 (13.6)	21.3 (10.3)	20.9 (17.2)	22.5 (12.2)	21.0 (16.3)	23.3 (15)
BUN (mg/dL)	12.8 (3.8)	13.0 (3.4)	13.4 (3.8)	12.1 (2.6)	13.8 (3.1)	11.9 (3.3)
Creatinine (mg/dL)	0.7 (0.1)	0.7 (0.1)	0.7 (0.1)	0.7 (0.1)	0.7 (0.2)	0.7 (0.1)
Na (mEq/L)	140.6 (2.0)	140.0 (1.7)	140.6 (1.5)	140.5 (1.7)	140.7 (1.7)	140.9 (1.8)
Cl (mEq/L)	104.4 (1.1)	104.0 (2.5)	103.5 (1.3)	103.2 (2.3)	103.4 (1.5)	103.8 (2.3)
K (mEq/L)	4.2 (0.3)	4.3 (0.4)	4.5 (0.4)	4.4 (0.3)	4.3 (0.4)	4.2 (0.2)
Total cholesterol (mg/dL)	201.6 (27.3)	207.8 (27.1)	206.1 (29.4)	211.5 (25.6)	219.2 (33.4)	215.8 (27.3)
LDL-cholesterol (mg/dL)	118.8 (27.0)	125.0 (25.5)	122.1 (27.4)	126.1 (26.7)	130.4 (26.8)	129.1 (25.9)
HDL-cholesterol (mg/dL)	64.5 (18.2)	63.2 (10.8)	66.6 (19.2)	65.4 (10.9)	69.9 (22.1)	68.1 (14.2)
TG (mg/dL)	83.0 (43.2)	87.0 (44.2)	92.5 (46.9)	104.8 (68.8)	98.4 (69.4)	100.7 (51.1)
FPG (mg/dL)	86.0 (5.5)	87.0 (7.2)	84.4 (7.6)	85 (7.8)	84.3 (7.3)	84.5 (7.5)
HbA1c (%)	5.3 (0.2)	5.3 (0.2)	5.3 (0.2)	5.4 (0.2)	5.4 (0.2)	5.4 (0.2)

Data are represented as mean (SD); body mass index (BMI), systolic blood pressure (SBP), diastolic blood pressure (DBP), white blood cell (WBC) count, red blood cell (RBC) count, hemoglobin (Hb), hematocrit (Ht), aspartate aminotransferase (AST), alanine aminotransferase (ALT), lactate dehydrogenase (LDH), alkaline phosphatase (ALP), γ-glutamyl trans-peptidase (γGTP), blood urea nitrogen (BUN), sodium (Na), chlorine (Cl), potassium (K), triglyceride (TG), fasting plasma glucose concentration (FPG), and glycated hemoglobin (HbA_{1c}).
